# Anomalous Left Circumflex Origin From Right Pulmonary Artery: Concern for Coronary Steal Revealed by Multimodal Imaging

**DOI:** 10.1016/j.jaccas.2025.104204

**Published:** 2025-06-25

**Authors:** Girish Pathangey, Mohamed A. Allam, Mahmoud H. Abdelnabi, Ramzi Ibrahim, Patrick Sarkis, Sachin Pathangey, Kristen Sell-Dottin, Chadi Ayoub, Francois Marcotte, Hemalatha Narayanasamy

**Affiliations:** a Department of Cardiovascular Disease, Mayo Clinic, Phoenix, Arizona, USA; b Department of Advanced Heart Failure and Transplant, Mayo Clinic, Phoenix, Arizona, USA; c Oakland University William Beaumont School of Medicine, Royal Oak, Michigan, USA; d Department of Cardiothoracic Surgery, Mayo Clinic, Phoenix, Arizona, USA

**Keywords:** cardiac magnetic resonance, cardiomyopathy, computed tomography, coronary angiography, coronary vessel anomaly, hybrid imaging, myocardial ischemia, nuclear medicine, perfusion, positron emission tomography

## Abstract

**Background:**

Coronary artery anomalies occur in 0.03% to 0.28% of the population, with anomalous left circumflex artery from right pulmonary artery (AoLCx-RPA) a rare subset associated with increased ischemia risk and cardiac death. Early recognition and functional assessment are essential for optimal management given limited guidelines.

**Case Summary:**

We present a 43-year-old man with no significant history who developed 1-month exertional dyspnea, electrocardiographic changes, and elevated troponin concerning for non–ST-segment elevation myocardial infarction. Coronary angiography revealed nonobstructive arteries with retrograde filling via right coronary artery collaterals, suggestive of anomalous left circumflex artery. Multimodal imaging—including computed tomography angiography, cardiac magnetic resonance, and positron emission tomography—confirmed AoLCx-RPA with ischemia, prompting surgical correction.

**Discussion:**

This case highlights diagnostic and treatment pathways for AoLCx-RPA, contributing to limited evidence. We discuss current guidelines, multimodal imaging's role in evaluating anatomic and functional significance, and advanced hybrid imaging's utility in management.

**Take-Home Message:**

Clinical suspicion for coronary anomalies may warrant multimodal imaging and multidisciplinary approach for risk stratification and outcome optimization.

## Graphical Abstract

**Figure:**
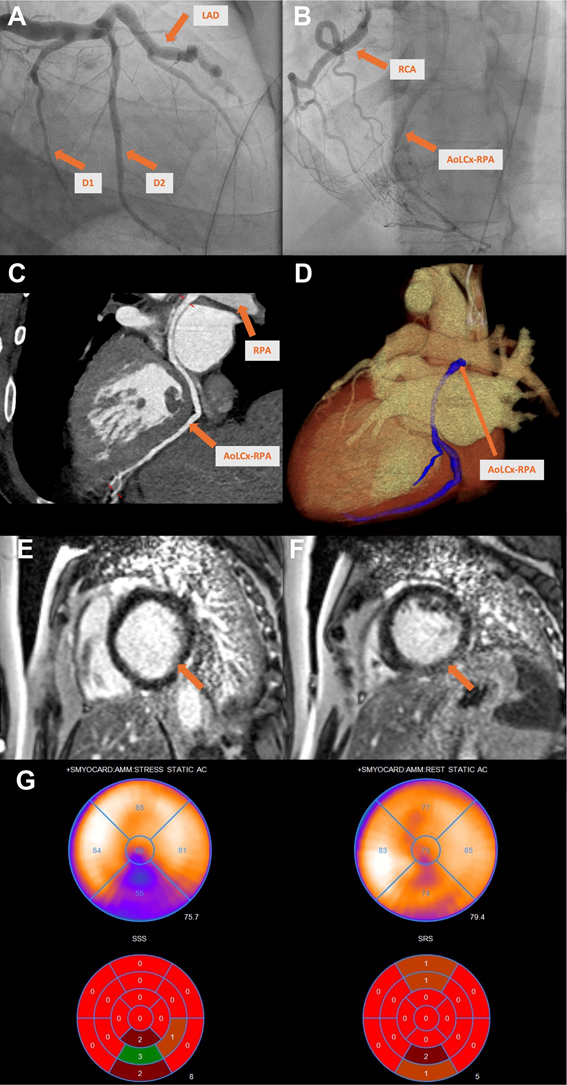

**Visual Summary:**
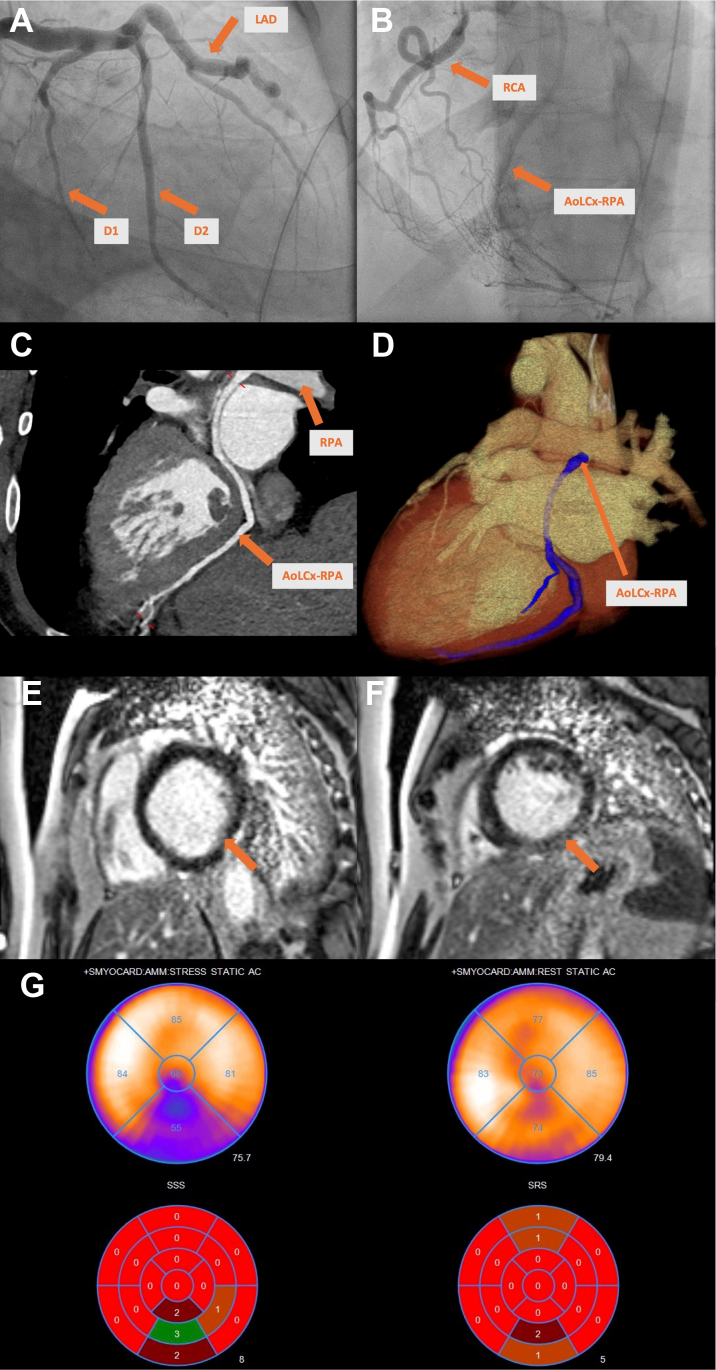
Anomalous Left Circumflex Artery From Right Pulmonary Artery: Multimodal Imaging Overview (A) Coronary angiogram (RAO caudal): dilated LAD with absent LCx. (B) Coronary angiogram (LAO cranial): RCA collaterals and delayed LCx filling. (C and D) Cardiac CT: LCx originating from RPA (2.3 cm from bifurcation) with fistulous connections to LAD and RCA. (E) cMRI: subendocardial delayed enhancement in basilar inferior wall. (F) cMRI: delayed enhancement, mid-apical lateral, inferior LV walls. (G) PET-CT (stress, rest): severe reversible ischemia (18% myocardium) from basal-apical, inferior wall with mild hypokinesis inferior segments during stress. EF: prestress 55%, poststress 54%, TID ratio 1.06. Lowest perfusion in RCA territory.

Coronary artery anomalies are present in 0.03% to 0.28% of the population, with an anomalous left circumflex artery from the right pulmonary artery (AoLCx-RPA) being exceptionally rare.^1^ A 2020 meta-analysis reported AoLCx-RPA as 14 times less frequent than the anomalous left main coronary artery from the pulmonary artery (ALCAPA), which itself has a prevalence of 0.008%.^2^ Given its rarity, there are no established guidelines for its management, unlike more common anomalies. This case presents a diagnostic approach and treatment pathway for AoLCx-RPA, emphasizing the role of multimodal imaging in elucidating its pathophysiology and the utility of advanced functional and hybrid imaging in management, thereby contributing to limited body of evidence that may guide potential approaches to rare coronary anomalies.

## History of Presentation

**Figure 1:**
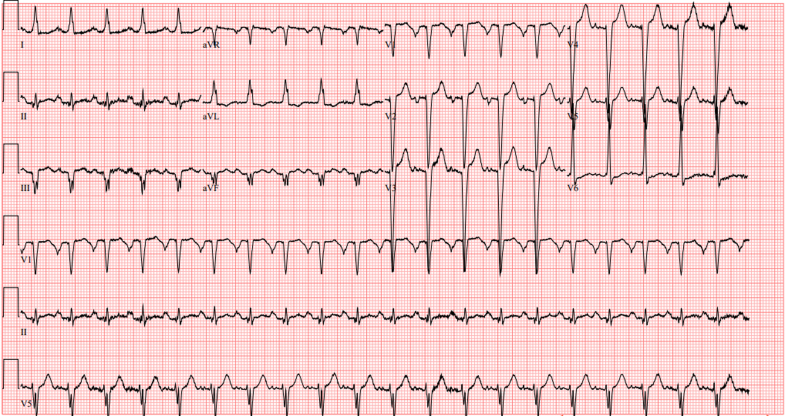
ECG on Admission Initial electrocardiogram on admission: sinus tachycardia (122 beats/min), PR interval of 164 milliseconds, QRS duration of 108 milliseconds, normal axis, inferior Q waves in inferior leads, and nonspecific ST-segment/T-wave changes in anterolateral leads.

A 43-year-old man with no significant history presented to clinic with 1-month worsening dyspnea. Electrocardiogram raised concern for non–ST-segment elevation myocardial infarction with nonspecific ST-segment/T-wave changes and inferior Q waves (Figure 1), prompting aspirin loading and transfer to a tertiary hospital. On arrival, the patient was hemodynamically stable but diaphoretic, tachycardic (121 beats/min), hypertensive (182/139 mm Hg), and had an saturation of peripheral oxygen of 99% on 2 L oxygen. Physical examination revealed jugular venous distention with cold extremities and bilateral ankle edema.

Initial diagnostics showed elevated high-sensitivity troponin T (194, peaking at 246 ng/L), N-terminal pro–B-type natriuretic peptide (9570 pg/mL), D-dimer (1740 ng/mL), and creatinine (1.49 mg/dL). Computed tomography pulmonary angiography excluded pulmonary embolism but revealed bilateral pulmonary edema. Transthoracic echocardiogram (TTE) demonstrated severe global left ventricular (LV) dysfunction (ejection fraction [EF]: 15%) with regional variability. A 2.4- × 2.4-cm mobile inferobasal LV thrombus was identified, along with elevated LV filling pressures and right ventricular (RV) systolic pressure of 55 mm Hg, and dilated inferior vena cava. The patient was initiated on heparin, nitroglycerin drip, intravenous diuretics, and dobutamine for inotropic support.

## Past Medical History

The patient had no family history of premature coronary disease, and no personal history of diabetes, hypertension, dyslipidemia, or other systemic disease. The patient denied tobacco or illicit drug use, with occasional alcohol intake.

## Differential Diagnosis

Differential included cardiogenic shock secondary to type 1 or type 2 non–ST-segment elevation myocardial infarction, myocarditis, and hypertensive or genetic cardiomyopathy.

## Investigations

**Figure 2:**
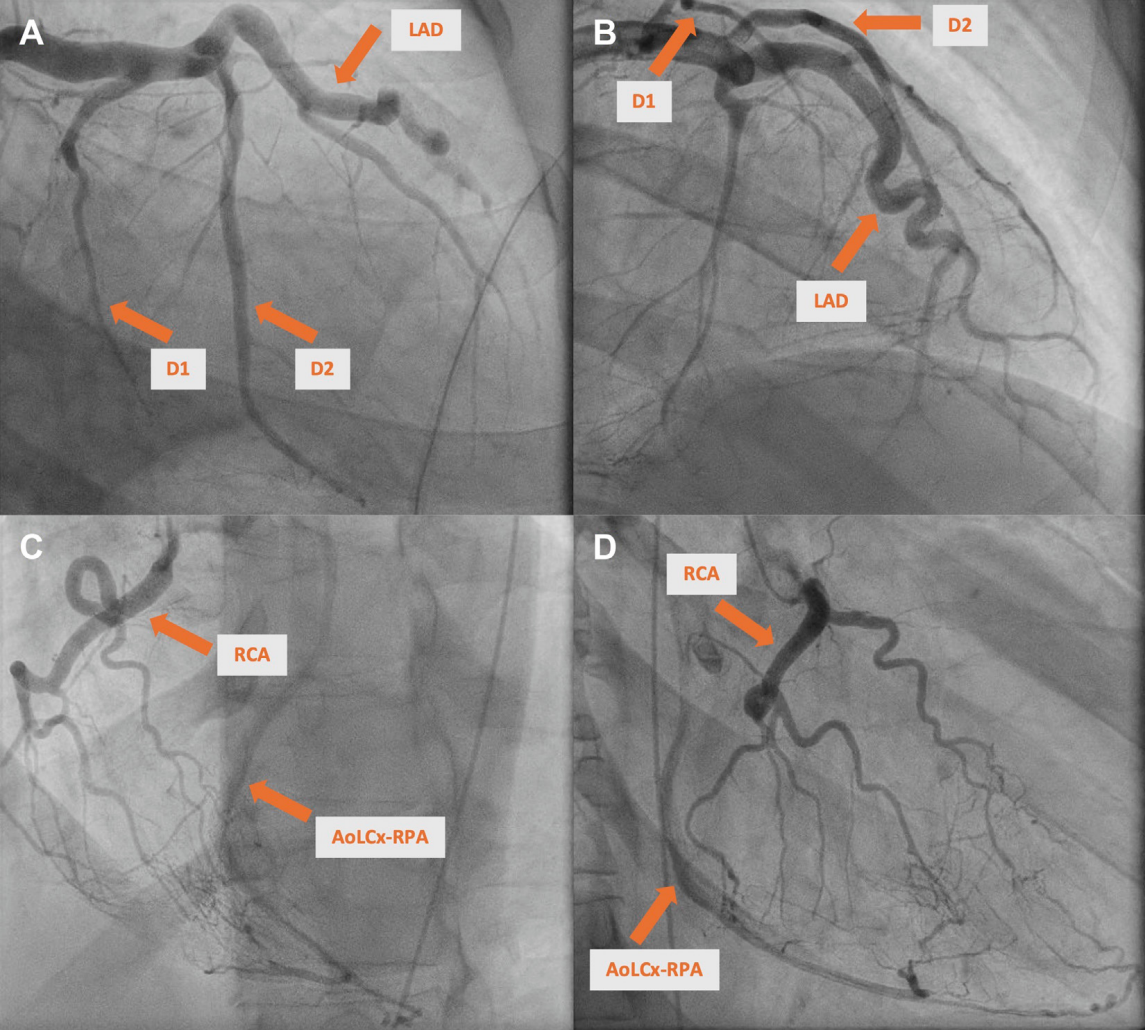
Coronary Angiogram (A) (RAO caudal) and (B) (AP cranial): prominent, dilated LAD with absence of the left circumflex artery (LCx) and notable septal perforators. Video 1 highlights LAD collateralization to the RCA. (C) (LAO cranial) and (D) (RAO cranial): RCA collaterals with delayed filling of the anomalous LCx, further demonstrated in Video 2. AoLCx-RPA = anomalous left circumflex artery from the right pulmonary artery; AP = anteroposterior; D1 = first diagonal branch; D2 = second diagonal branch; LAO = left anterior oblique; LAD = left anterior descending artery; RAO = right anterior oblique; RCA = right coronary artery.

**Figure 3:**
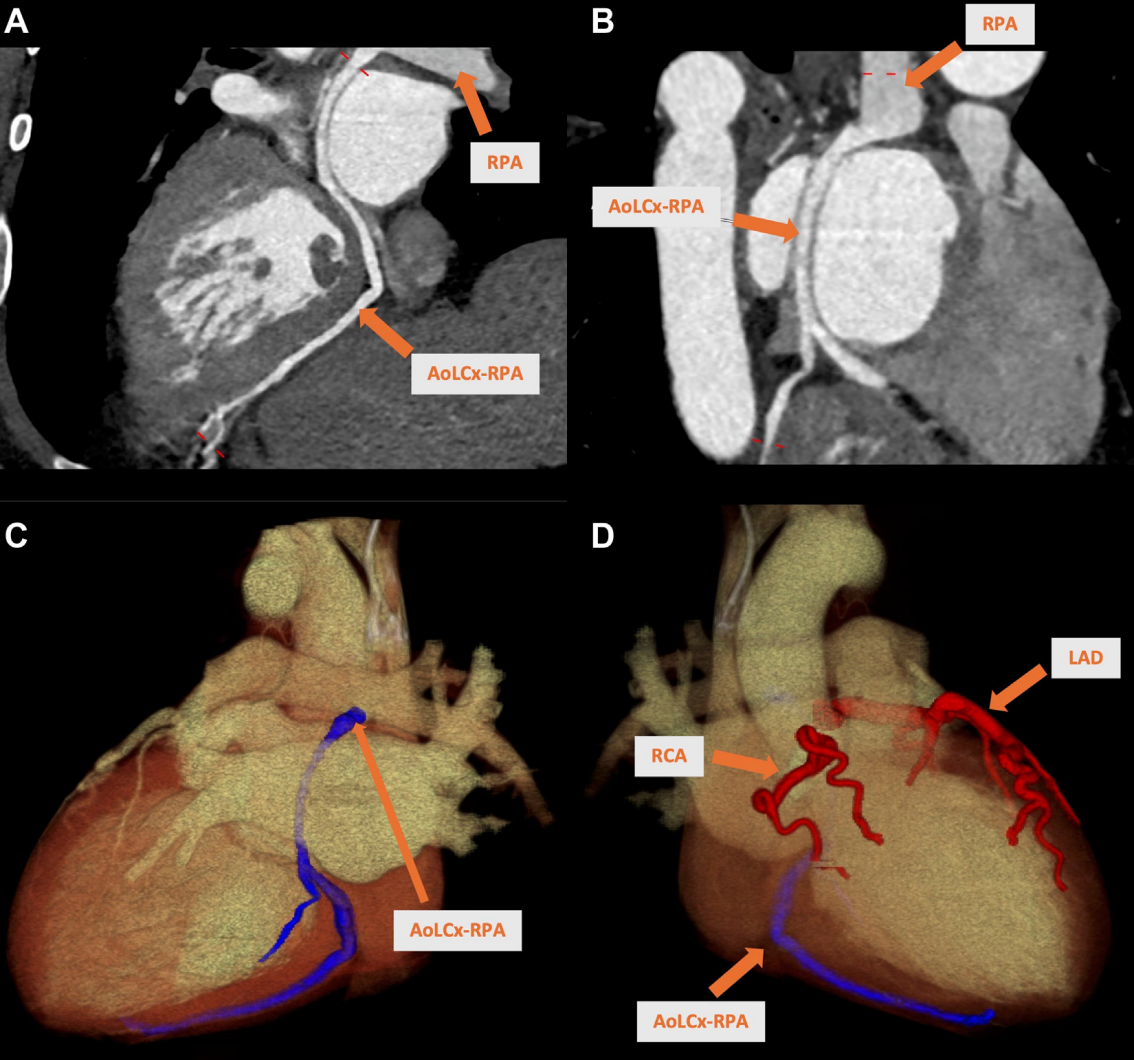
Cardiac CT Images Cardiac computed tomography images (A and B) and 3-dimensional segmentation (C and D) show anomalous origin of the LCx from the right PA, approximately 2.3 cm from the PA bifurcation, with multiple fistulous communications to the LAD and RCA. LAD and RCA have normal aortic origins, with the LAD appearing prominently tortuous (up to 8 mm proximally) and the proximal RCA measuring up to 6 mm. No coronary plaque or stenosis is observed. AoLCx-RPA = anomalous left circumflex artery from the right pulmonary artery; LAD = left anterior descending artery; PA = pulmonary artery; RCA = right coronary artery.

Coronary angiography revealed left-dominant circulation with nonobstructive, dilated coronary arteries, arteriovenous communications, and prominent collaterals. Retrograde filling via right coronary artery (RCA) collaterals suggested anomalous left circumflex artery (LCx) origin from right pulmonary artery (RPA) (Figure 2, Videos 1 and 2). Right heart catheterization indicated normal pressures but low cardiac output (cardiac index: 1.3 L/min/m^2^ by Fick, 1.1 L/min/m^2^ by thermodilution) and pulmonary vascular resistance of 2.1 Wood units. Coronary computed tomography angiography confirmed LCx originated anomalously from RPA, 2.3 cm from pulmonary artery bifurcation, with multiple fistulous connections to left anterior descending artery (LAD) and RCA (Figure 3, Videos 3 and 4).

**Figure 4:**
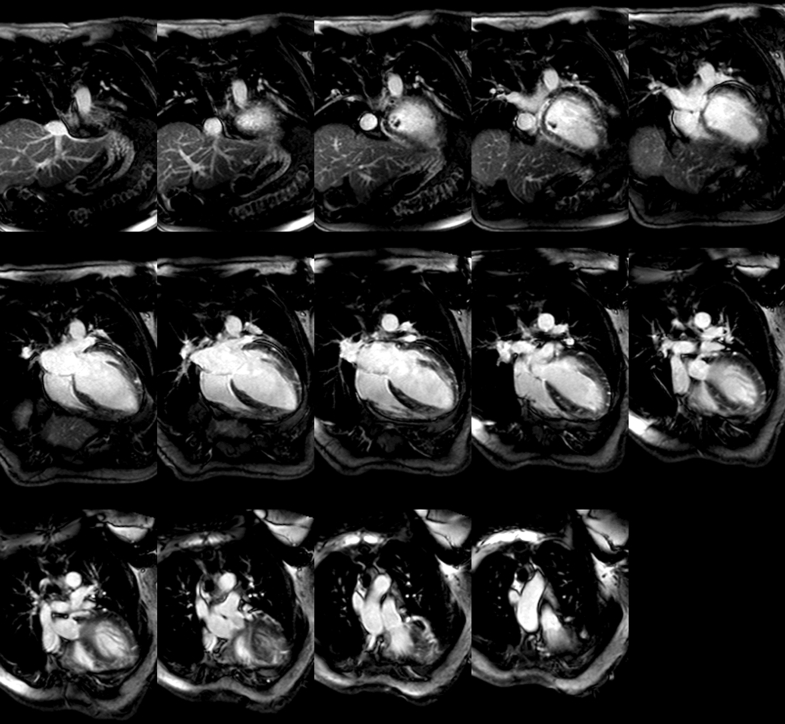
Inpatient cMRI (LGE GRE 4-Chamber) Inpatient cardiac magnetic resonance (late gadolinium enhancement [LGE] GRE 4-chamber) demonstrates severe left ventricular (LV) dilatation (end-diastolic volume: 183 mL/m^2^) and marked systolic dysfunction (LV ejection fraction [EF]: 16%), with 25% wall-thickness LGE in the basal/midinferior walls and patchy nonischemic LGE in the subepicardial/midlateral walls. A 2.1- × 1.0- × 1.6-cm nonenhancing sessile thrombus is present. Right ventricular (RV) size and function (RV EF: 50%) are preserved, with trivial inferoposterior pericardial effusion and no pleural effusion. GRE = gradient recalled echo.

**Figure 5:**
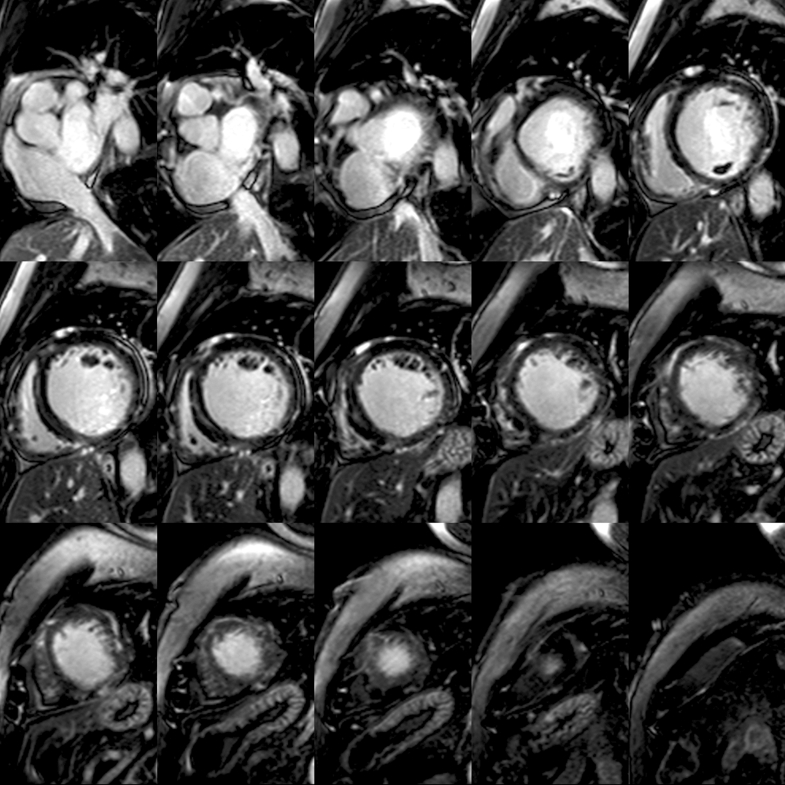
Inpatient cMRI (LGE GRE Short-Axis) Inpatient cardiac magnetic resonance (late gadolinium enhancement [LGE] GRE short-axis) demonstrates 25% wall-thickness LGE in basal/midinferior walls, patchy subepicardial/midlateral LGE, and a 2.1- × 1.0- × 1.6-cm nonenhancing sessile thrombus. Abbreviation as in Figure 4.

Cardiac magnetic resonance (cMRI) showed severe LV dilatation (end-diastolic volume: 183 mL/m^2^) and systolic dysfunction (LV EF: 16%) with subendocardial basal and midinferior late gadolinium enhancement (LGE) affecting 25% wall thickness. Patchy nonischemic subepicardial and midlateral LGE, overlying nonenhancing 2.1- × 1.0- × 1.6-cm sessile thrombus, was also observed. RV size and systolic function were normal (RV EF 50%) with trivial inferoposterior pericardial effusion without pericardial LGE (Figures 4 and 5).

## Management

Despite inotropic support, guideline-directed medical therapy (GDMT) optimization was limited by hypotension, and the course was complicated by recurrent nonsustained ventricular tachycardia. A single-lead implantable cardioverter-defibrillator (ICD) was implanted for primary prevention, and the patient was listed for heart transplantation (United Network for Organ Sharing status 6). At discharge, warfarin and beta-blocker were prescribed, with GDMT titrated in outpatient follow-up.

**Figure 6:**
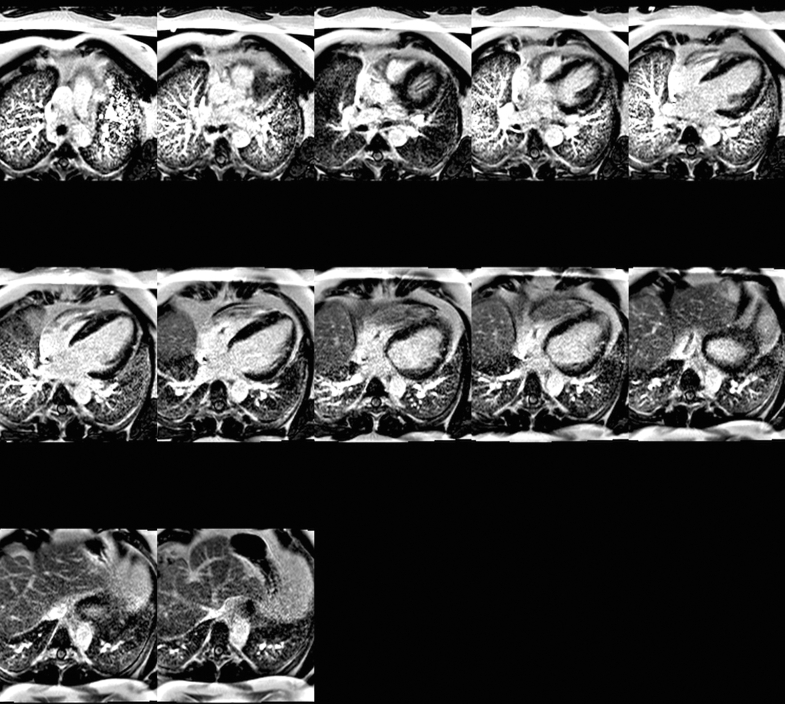
Post-Discharge cMRI (LGE WB 4-Chamber) Postdischarge cardiac magnetic resonance (late gadolinium enhancement WB 4-chamber) reveals stable, scattered nonvascular delayed enhancement in the left ventricle, including subendocardial enhancement in the basilar inferior wall and midapical lateral/inferior walls. Q_p_/Q_s_ of 1.18 indicates no significant left-to-right shunt through the anomalous left circumflex artery from the right PA. Previously noted thrombus along the basilar inferior left ventricular wall has resolved. PA = pulmonary artery; WB = white blood.

**Figure 7:**
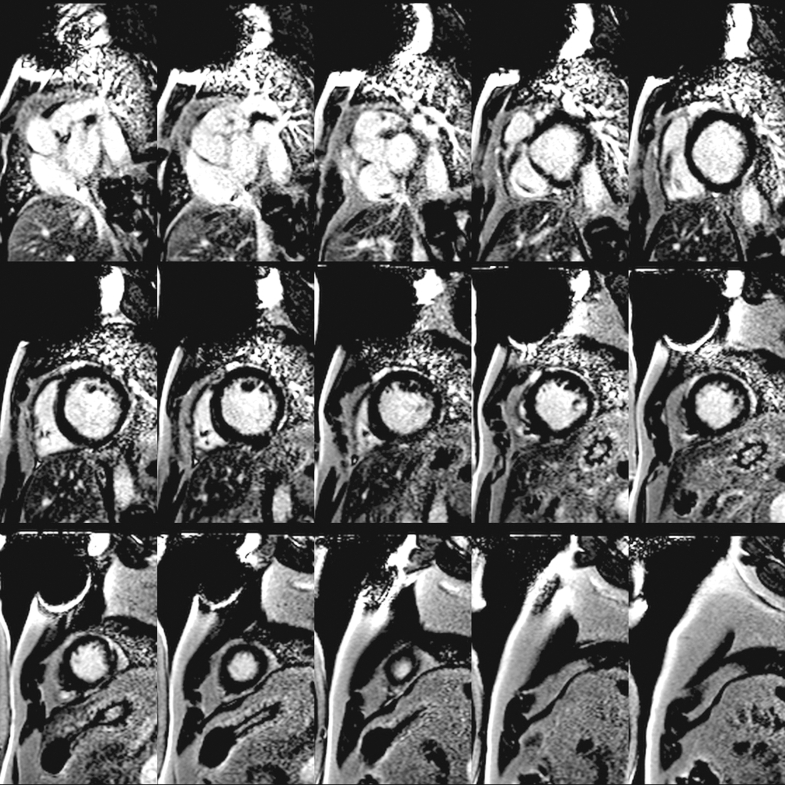
Post-Discharge cMRI (LGE WB Short-Axis) Postdischarge cardiac magnetic resonance (late gadolinium enhancement WB short-axis) demonstrates stable, scattered nonvascular delayed enhancement in the left ventricle with subendocardial enhancement in the basilar inferior wall and midapical lateral/inferior walls. WB = white blood.

Two months postdischarge, TTE showed thrombus resolution but persistent LV dysfunction (EF: 20%). Six months postdischarge, cMRI revealed improved EF of 40% with mild nonischemic LGE in the mid-to-apical lateral-inferior walls (<5% myocardial mass) and moderate mitral regurgitation (MR) (Figures 6 and 7).

**Figure 8:**
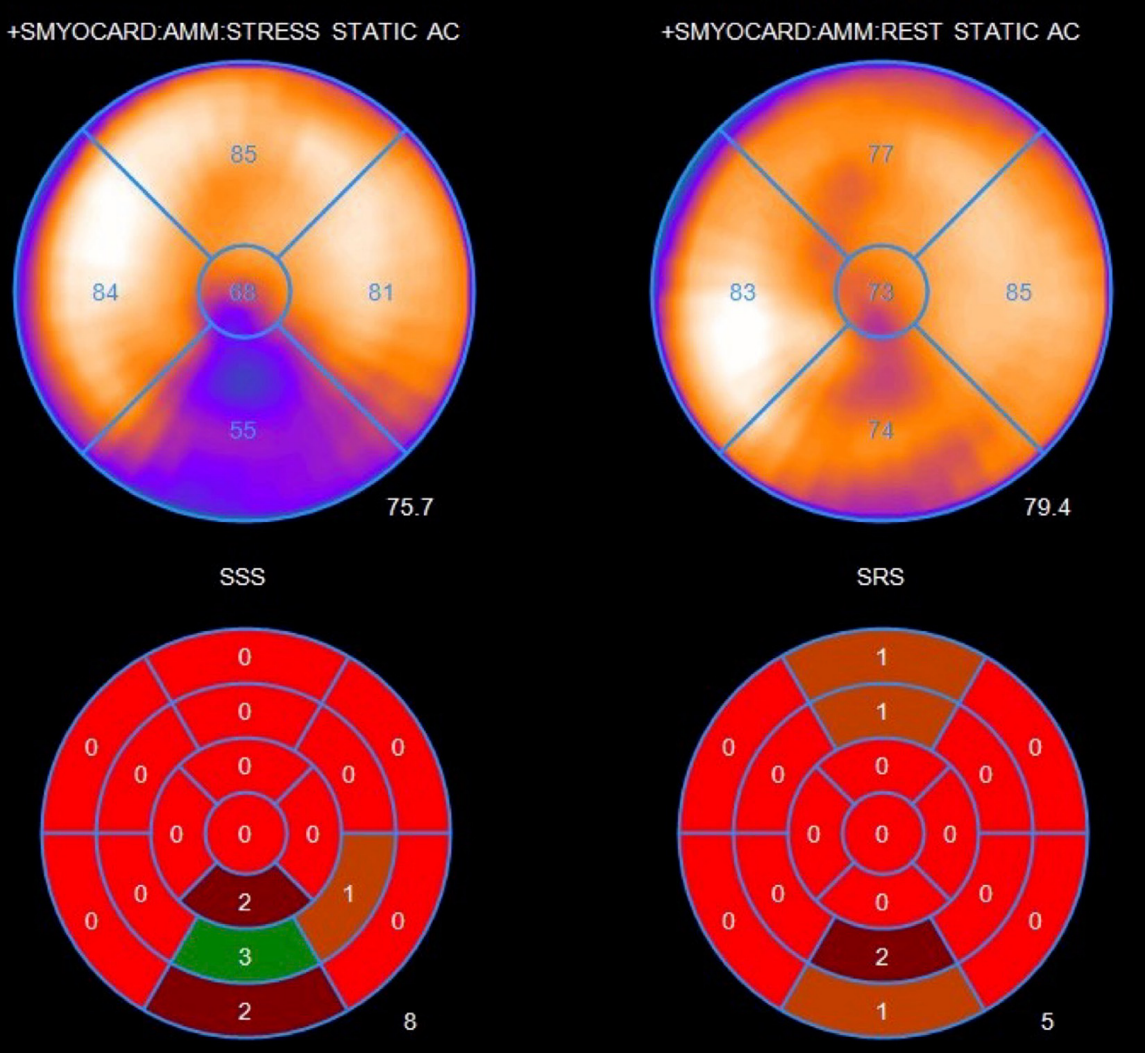
Post-Discharge Cardiac PET-CT Stress Test Postdischarge positron emission tomography-computed tomography (stress and rest) revealed medium-sized, severely dense, reversible ischemia involving 18% of the myocardium, extending from the basal to apical inferior wall, with lowest perfusion noted in right coronary artery territory.

Positron emission tomography-computed tomography (PET-CT) revealed medium-sized, dense reversible perfusion defect in the basal-to-apical inferior wall (18% myocardium), indicating ischemia. Mild hypokinesis was observed in inferior segments, with prestress and poststress EF of 55% and 54%, respectively, and transient ischemic dilation ratio of 1.06, suggesting lowest perfusion in the RCA territory (Figure 8, Supplemental Figure 1).

## Outcome and Follow-Up

The patient's transplant listing was downgraded due to clinical improvement on GDMT. At outpatient follow-up, a multidisciplinary team evaluated potential interventions, including LCx ligation with coronary artery bypass grafting or LCx reimplantation to the ascending aorta.

**Figure 9:**
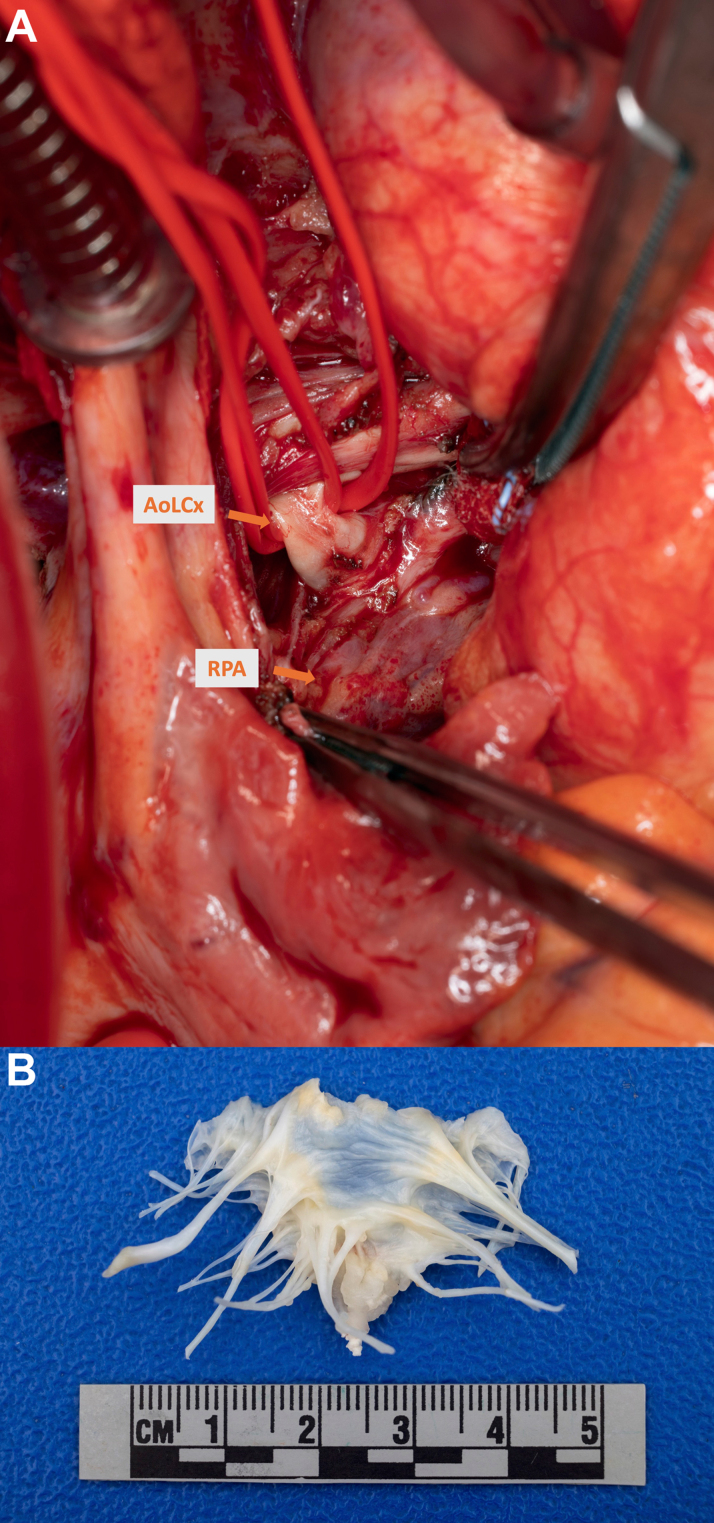
Intraoperative Images (A) Anomalous coronary artery (AoLCx) originating from the right pulmonary artery (RPA). (B) Resection of the native mitral valve showing endocardial vegetation.

The course was complicated by *Streptococcus lutetiensis* endocarditis, likely secondary to dental caries. Readmission TTE showed EF of 66%, moderate to severe MR with 0.8 × 0.5 cm mobile mitral valve vegetation, and possible echodensity on the ICD lead. Transesophageal echocardiogram confirmed mitral valve endocarditis with a 13 × 10 × 6 mm vegetation, severe MR, and ICD lead sessile mass with preserved LV EF. Due to active infection requiring definitive source control and ischemia correction, a shared decision-making process with the patient and family led to mitral valve replacement (27/29 mm On-X mechanical valve, Artivion, Inc) after debridement and single-chamber ICD removal (without reimplantation given EF recovery), left atrial appendage ligation with a 40 mm AtriCure ProV clip (AtriCure, Inc), and LCx reimplantation to the aorta via short saphenous vein interposition graft (Figure 9).

## Discussion

AoLCX-RPA is an exceedingly rare coronary anomaly that presents considerable diagnostic and management challenges. Unlike more commonly encountered coronary anomalies, AoLCX-RPA is associated with increased risk of ischemia, myocardial dysfunction, and sudden cardiac death due to coronary steal and impaired myocardial perfusion.^3^^,^^4^

Advanced imaging modalities are pivotal in characterizing anatomic and functional aspects of complex coronary anomalies. We used computed tomography angiography and cMRI to identify the anomalous LCx origin as recommended by American College of Cardiology/American Heart Association congenital heart disease guidelines (Class I, Level of Evidence: C).^3^^,^^5^ Conversely, although coronary angiography is informative, it holds a Class IIa recommendation due to its limited ability to define the initial coronary course, with studies reporting 41% failure rate in achieving complete anatomic visualization.^5^

In cases where coronary angiography and magnetic resonance imaging are inconclusive in assessing shunting potential, functional imaging remains integral to evaluating the clinical significance of coronary anomalies; however, the optimal modality for ischemia detection remains debated. Exercise electrocardiogram and stress echocardiography, although widely accessible, are often limited by operator variability and absence of standardized protocols.^3^ Emerging advanced and hybrid modalities, such as PET-CT, single-photon emission computed tomography-computed tomography, and stress cMRI, have shown promise for identifying myocardial perfusion deficits and ischemic burden in complex anatomy, thereby aiding therapeutic decision-making.^3^^,^^5^^,^^6^

In this patient, PET-CT revealed an 18% myocardial perfusion defect with hypokinesis in the inferior LV segments during stress, likely indicating coronary steal via RCA collaterals to the AoLCX-RPA and resulting in under perfusion and myocardial dysfunction.^7^ Limited institutional experience and lengthy protocol times precluded option of stress cMRI; however, it remains a valuable radiation-free option offering higher spatial and contrast resolution than PET-CT—while also enabling comprehensive assessment of wall motion, ischemia, and infarction in a single study. Moreover, it can detect small perfusion defects and evaluate viability.^6^ Nonetheless, PET-CT provides more established prognostic data for surgical planning in coronary anomalies, with chest pain (OR: 9.73; *P* < 001) and ischemia (OR: 6.79; *P* = 0.002) significantly correlating with surgical management.^8^

Management of AoLCX-RPA remains challenging due to absence of specific guidelines, in contrast to more common anomalies such as ALCAPA, for which surgery is often recommended even in asymptomatic patients (American Heart Association/American College of Cardiology Class I, Level of Evidence: B; European Society of Cardiology Class I, Level of Evidence: C). Recommended surgical approaches for ALCAPA include direct reimplantation into the aorta when feasible or use of an interposition graft (eg, saphenous vein) if reimplantation is limited by adequate intramural segment. If neither approach is technically feasible, coronary artery bypass grafting with closure or ligation of the anomalous origin—typically using a mammary artery or saphenous vein conduit—may be performed.^3^

Current treatment strategies for AoLCx-RPA are determined on a case-by-case basis, guided by clinical presentation, ischemic burden, and imaging findings, ranging from conservative management to surgical revascularization based on patient-specific ischemia burden and symptoms.^2^, ^3^, ^4^ For this patient, initial management prioritized medical optimization, focusing on LV thrombus resolution with warfarin due to its established efficacy over new oral anticoagulants/direct oral anticoagulants in reducing thromboembolic risk.^9^ Furthermore, GDMT and ICD placement were implemented to reduce postoperative mortality and sudden cardiac death risk before surgical intervention, aligning with 2008 Heart Rhythm Society guidelines (Class IIa; Level of Evidence: C) for high-risk patients exhibiting reduced EF and arrhythmia.^10^ Despite complications from endocarditis, the patient underwent surgical correction on EF recovery with removal of LCx from RPA and reimplantation to the aorta via short saphenous vein interposition graft, restoring systemic blood flow to LCx and improving myocardial perfusion.

Significant knowledge gaps persist in optimizing management for coronary anomalies, especially concerning the balance between anatomic and functional assessment for accurate risk stratification and treatment guidance. Further investigation is warranted to refine medical and surgical approaches for rare types like AoLCX-RPA due to their varied clinical and anatomic presentations. Establishing standardized terminology and a comprehensive classification of coronary anomalies is essential for improving clinical outcomes, supported by key registries such as the Congenital Heart Surgeons' Society Registry (outcomes in young patients), Anomalous Coronary Arteries Registry (treatment strategies in older patients), and the Sudden Death in the Young Registry (linking to sudden cardiac death).^5^

## Conclusions

This case underscores the importance of multimodal imaging in diagnosing and managing rare AoLCX-RPA, emphasizing comprehensive anatomic and functional assessments for surgical guidance. However, limited evidence and specific guidelines for rare coronary anomalies necessitate further research and risk-stratified protocols. Establishing standardized classifications and guidelines could enhance patient-centered, evidence-based management of these uncommon conditions.

## Funding Support and Author Disclosures

The authors have reported that they have no relationships relevant to the contents of this paper to disclose.

**Take-Home Messages**

• Coronary artery anomalies are infrequent, occurring in 0.03% to 0.28% of the population, with AoLCx-RPA being especially rare—14 times less common than ALCAPA, which has a prevalence of 0.008%.
• No established guidelines exist for the treatment of AoLCx-RPA; management is determined by clinical presentation, ischemic burden, and multimodal imaging findings, with options ranging from conservative management to surgical revascularization.
• Multimodal imaging provides valuable insights into the pathophysiology of the coronary steal effect in coronary artery anomalies.

## Appendix

Video 1 (Anteroposterior Cranial): Prominent, Dilated LAD With Absent Left Circumflex Artery and Notable Septal Perforators, With LAD Collateralization to the Right Coronary Artery”; video 2 caption as “(Right Anterior Oblique Cranial): Right Coronary Artery Collaterals With Delayed Filling of the Anomalous Left Circumflex Artery LAD = left anterior descending artery.

Video 2 (RAO Cranial): Right Coronary Artery Collaterals With Delayed Filling of the Anomalous Left Circumflex Artery

Video 3 Cardiac Computed Tomography in the Axial Plane

Video 4 Cardiac Computed Tomography in the Coronal Plane

Supplemental Figure 1 Stress PET-CT With Myocardial Flow Reserve Analysis
