# Supplementary material for: Anomalous Left Circumflex Origin From Right Pulmonary Artery: Concern for Coronary Steal Revealed by Multimodal Imaging
Source: JACC Case Rep. 2025 Jun 25;30(16):104204. doi: 10.1016/j.jaccas.2025.104204 (PMC12273817; doi:10.1016/j.jaccas.2025.104204)
Supplement: Supplemental Figure 1 — Stress PET-CT With Myocardial Flow Reserve Analysis [file mmc5.docx]

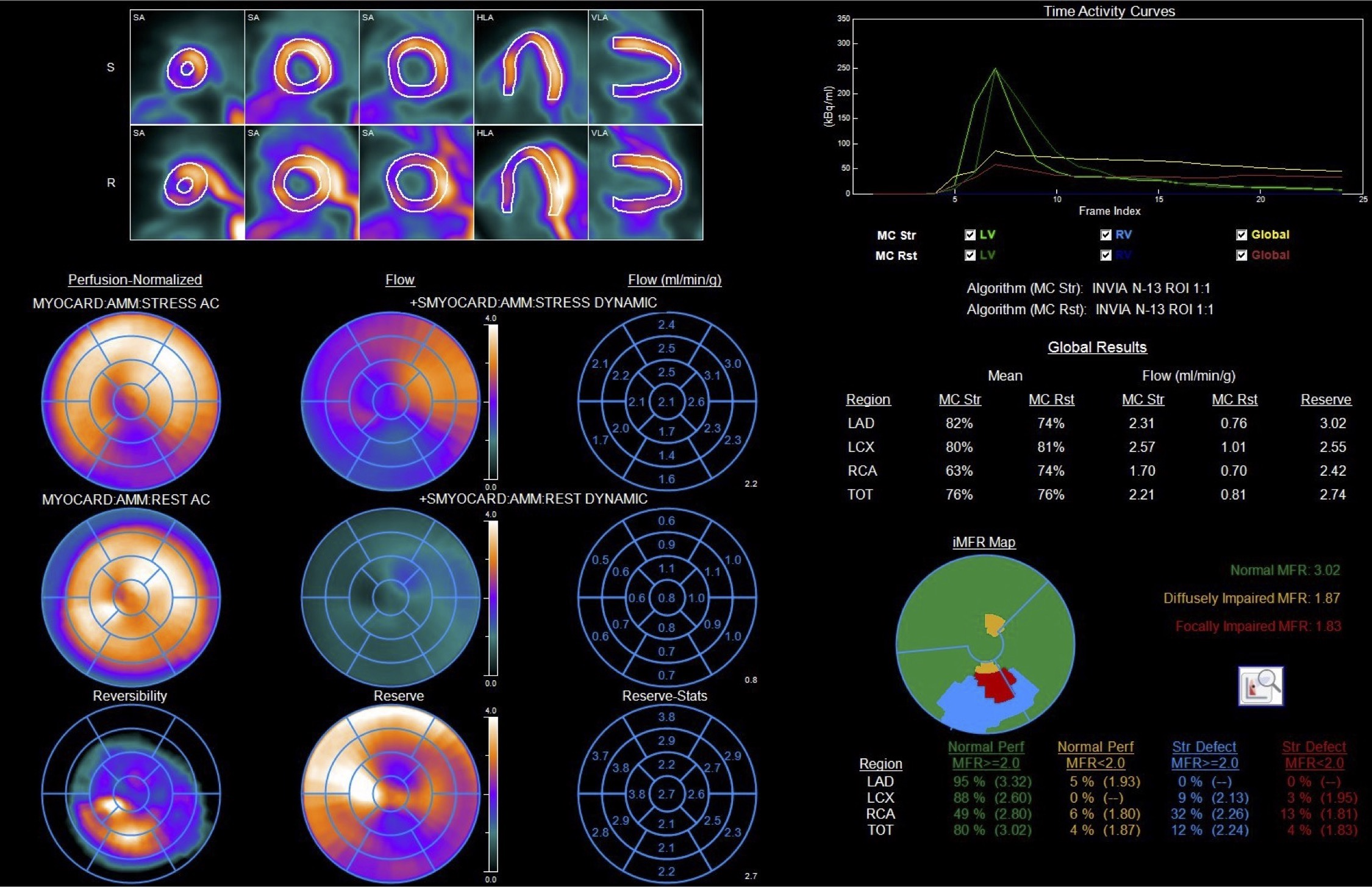


**Supplementary Image 1:** Post-discharge PET-CT showed quantitative flow reserves lowest in RCA territory at stress, with mild hypokinesis in inferior segments during stress. EF: 55% pre-stress, 54% post-stress; TID ratio: 1.06.
